# Microbiomes in a manganese oxide producing ecosystem in the Ytterby mine, Sweden: impact on metal mobility

**DOI:** 10.1093/femsec/fiaa169

**Published:** 2020-08-20

**Authors:** Susanne Sjöberg, Courtney W Stairs, Bert Allard, Felix Homa, Tom Martin, Viktor Sjöberg, Thijs J G Ettema, Christophe Dupraz

**Affiliations:** Department of Geological Sciences, Stockholm University, Svante Arrhenius väg 8, SE-106 91 Stockholm, Sweden; Department of Cell and Molecular Biology, Science for Life Laboratory, Uppsala University, Box 596, SE-751 23 Uppsala, Sweden; Man-Technology-Environment Research Centre (MTM), Örebro University, SE-701 82 Örebro, Sweden; Department of Cell and Molecular Biology, Science for Life Laboratory, Uppsala University, Box 596, SE-751 23 Uppsala, Sweden; Laboratory of Microbiology, Department of Agrotechnology and Food Sciences, Wageningen University, Stippeneng 4, 6708WE Wageningen, The Netherlands; Department of Cell and Molecular Biology, Science for Life Laboratory, Uppsala University, Box 596, SE-751 23 Uppsala, Sweden; Man-Technology-Environment Research Centre (MTM), Örebro University, SE-701 82 Örebro, Sweden; Department of Cell and Molecular Biology, Science for Life Laboratory, Uppsala University, Box 596, SE-751 23 Uppsala, Sweden; Laboratory of Microbiology, Department of Agrotechnology and Food Sciences, Wageningen University, Stippeneng 4, 6708WE Wageningen, The Netherlands; Department of Geological Sciences, Stockholm University, Svante Arrhenius väg 8, SE-106 91 Stockholm, Sweden

**Keywords:** Mn-oxidizers, birnessite, ecosystem, biofilms, shallow subsurface, REE fractionation, Ytterby mine

## Abstract

Microbe-mediated precipitation of Mn-oxides enriched in rare earth elements (REE) and other trace elements was discovered in tunnels leading to the main shaft of the Ytterby mine, Sweden. Defining the spatial distribution of microorganisms and elements in this ecosystem provide a better understanding of specific niches and parameters driving the emergence of these communities and associated mineral precipitates. Along with elemental analyses, high-throughput sequencing of the following four subsystems were conducted: (i) water seeping from a rock fracture into the tunnel, (ii) Mn-oxides and associated biofilm; referred to as the Ytterby Black Substance (YBS) biofilm (iii) biofilm forming bubbles on the Mn-oxides; referred to as the bubble biofilm and (iv) fracture water that has passed through the biofilms. Each subsystem hosts a specific collection of microorganisms. Differentially abundant bacteria in the YBS biofilm were identified within the *Rhizobiales* (e.g. *Pedomicrobium)*, PLTA13 *Gammaproteobacteria, Pirellulaceae, Hyphomonadaceae, Blastocatellia* and *Nitrospira*. These taxa, likely driving the Mn-oxide production, were not detected in the fracture water. This biofilm binds Mn, REE and other trace elements in an efficient, dynamic process, as indicated by substantial depletion of these metals from the fracture water as it passes through the Mn deposit zone. Microbe-mediated oxidation of Mn(II) and formation of Mn(III/IV)-oxides can thus have considerable local environmental impact by removing metals from aquatic environments.

## Teaser

Defining the spatial distribution of microorganisms and elements in this REE-enriched Mn-oxide producing ecosystem contribute to a better understanding of specific niches and parameters driving the emergence of these communities.

## INTRODUCTION

Metal contamination in aquatic environments is a major concern in many areas of the world due to adverse ecological effects. Microbial activity can limit the mobility of contaminants by producing highly reactive minerals with strong sorption capacities. A group of such reactive minerals are Mn-oxides (Nelson *et al*. 1999; Kay *et al*. 2001; Toner *et al*. 2006; Takahashi *et al*. 2007; Peña *et al*. 2010). Abiotic oxidation of Mn(II) in solution by O_2_ is thermodynamically favorable but reaction rates are slow (Morgan 2005; Luther 2010). Most Mn-oxidation in nature is therefore believed to be microbially driven (Tebo *et al*. 2004). These microbially produced Mn-oxides have substantially higher sorption capacities than their abiotic-synthetic analogs (Zhou, Kim and Ko 2015). Microbial oxidation of Mn(II) and subsequent formation of sparingly soluble oxides of Mn(III) and Mn(IV) can therefore have considerable local environmental impact and could have an important role in removal of Mn and trace metals from aquatic environments.

A porous dark substance, here denoted Ytterby Black Substance (YBS), was observed in 2012 exuding from fractures in an underground tunnel leading to the main shaft of the Ytterby mine, Sweden. It was established that the main component was a microbially mediated birnessite-type Mn-oxide with the assessed composition M_0.4–0.6_[Mn(III, IV)]_2_O_4_∙(H_2_O)_n_, where M was predominantly Ca, Mg and rare earth elements (REE) (Sjöberg et al. 2017, 2018). The REE fraction constituted as much as 10 000 ppm of the dry mass, but there were also a number of other elements associated with the birnessite phase. The microbially mediated formation of Mn-oxides in Ytterby is a relevant example of a natural system with high capacity to remove Mn(II) and scavenge trace elements from the local groundwater. Observations of similar environments driven by biological production of Mn-oxide precipitates are reported in Burkhardt *et al*. (2009), Akob *et al*. (2014), and Bohu *et al*. (2016).

**Figure 1.**
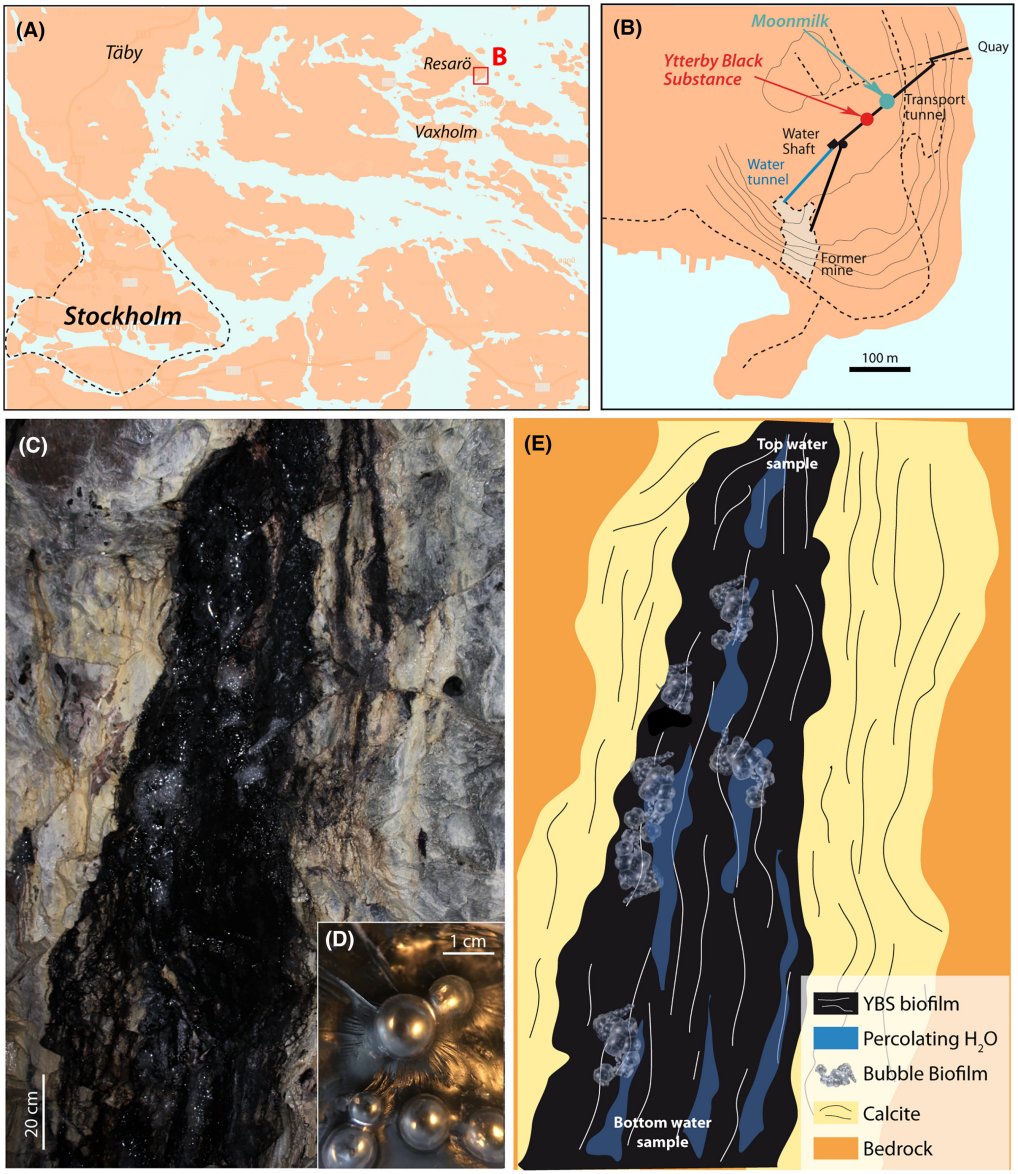
Maps showing the location of the Ytterby mine and the Mn deposit. **(A)**, Map of the Stockholm area with location of Resarö and the Ytterby mine indicated. **(B)**, Map of subterranean tunnels linking the Ytterby mine shaft with a more recently constructed quay to the NE. The Mn-oxides (referred to as the Ytterby black substance, YBS) precipitate from water provided by rock fractures that crop out in these tunnels. Modified from Swedish Fortifications Agency (2012). **(C)**, Photograph of the Mn deposit. **(D)**, Close-up of bubble biofilm. **(E)**, Sketch showing sampling locations, underlying lithified CaCO_3_ and bubble biofilm.

This study defines the spatial distribution of microorganisms and elements in the Mn-oxide producing ecosystem in order to better understand the formation of specific niches and parameters driving the emergence of these communities and associated metal accumulation. Along with elemental analyses, high-throughput sequencing of the following four subsystems were conducted: (i) water seeping from a rock fracture into the tunnel, (ii) Mn-oxides and associated biofilm (YBS biofilm) (iii) biofilm forming bubbles on the Mn-oxides (bubble biofilm) and (iv) fracture water that has passed through the biofilms down the approximately 2 m tall rock wall (Fig. 1). Fracture feed water from an adjacent system, not producing Mn-oxides, was also sampled for comparative purposes. Special focus is on determining the influence of the fracture water's elemental content and planktonic community composition for the emergence of the YBS biofilm and its differentially abundant taxa. Also, (i) biofilm cation binding capacity of aqueous Mn(II/III) and (ii) the simultaneous adsorption/co-precipitation of trace elements within the YBS biofilm were assessed by analyzing the water before and after it passed through the biofilms.

### Study site

The Ytterby mine is located on Resarö, an island along the Baltic Sea shore about 30 km NE of Stockholm (Fig. 1A). The Mn-oxide producing ecosystem is located in a tunnel leading to the main shaft of the former quartz and feldspar mine, also known for the discovery of Ta and seven of the REE (Nordenskjöld 1904; Enghag 1999). The tunnel system, which was constructed in the 1950s to convert the former mine into a fuel deposit for the Swedish Armed Forces, is approximately 400 m long and situated 29 m below ground surface and 5 m above Baltic Sea mean sea level (Fig. 1B). The tunnel goes through granitic and mafic rocks of varying chemical composition and metamorphic grade and links the old mine shaft with a quay used for loading and unloading the stored fuels (see Sjöberg 2012 and 2014 for more details on the geological setting). The majority of observed water-bearing fractures in this tunnel stretch are associated with Mn precipitates overlying a lithified layer of CaCO_3_, while only a few are associated with moonmilk deposits, a soft, not lithified rock wall coating of CaCO_3_ (Cailleau 2009).

Three different petroleum products were stored directly against the rock wall in the mine shaft (located approximately 200 m away from the studied Mn accumulations) over a 35–40 year period. Jet fuel during the 1950s and approximately 25 years afterwards, and more recently two types of diesel (J&W Energi och Miljö,Kemakta Konsult AB 2001). A continuous inflow of groundwater into the shaft, situated below the natural groundwater level, prevented the fuels from spreading into the rock. In 1995 the storage of petroleum products was brought to an end and it was emptied from diesel and closed down.

The studied Mn accumulations occur as rock wall coatings in a tunnel stretch located in the unsaturated zone, ca. 200 m from the mine shaft. Water-bearing rock fractures provide a continuous supply of water into the fully oxidized tunnel environment which holds a nearly constant temperature of 8°C year round (Fig. 1B and C). The seeping water provides elements and nutrients for two distinctively different biofilms to form: a Mn-oxide producing biofilm (hereafter referred to as the YBS biofilm) and an associated gas-trapping biofilm (hereafter referred to as the bubble biofilm and the subject of a separate paper; Sjöberg *et al*. 2020) (Fig. 1C and D). The maximum age of the Mn accumulation is 60–70 years, assuming that sequestration started when the tunnel was built. Artifical lighting is used during mine maintenance, on average 2–3 h/month in the otherwise dark tunnel.

## MATERIALS AND METHODS

### Sample collection

Samples for rRNA gene analyses were collected from three subsystems (the fracture water, the YBS biofilm and the bubble biofilm) of the Mn-oxide producing ecosystem. The fracture water was sampled at two locations (for rRNA gene as well as elemental analyses): at the top of the deposit, just as the water emerges from the fracture associated with the YBS precipitate (referred to as FW_YBS_T) and at the bottom of the deposit after it has passed through the YBS down the approximately 2 m tall rock wall (referred to as FW_YBS_B) (Fig. 1E). The YBS biofilm and bubble biofilm samples were collected from randomly chosen spots located in the area between the top and bottom of the rock wall. One YBS biofilm sample collected three years prior to the other samples, from the same area, was added (sample id. 512). Details on YBS sampling used for geochemical analyses, as well as on the elemental content of the YBS, are given in Sjöberg *et al*. (2017). Water from an adjacent fracture, not producing Mn-oxides, was also sampled for comparative purposes (located ca 50 m away in the same tunnel and associated with a moonmilk, CaCO_3_, deposit and referred to as FW_MM_T). All samples used for molecular analyses (except sample id. 512) were collected in February 2018.

Water samples for elemental analyses were filtered with polypropylene membrane syringe filters, 0.2 µm pore size (VWR) on site, acidified (HNO_3_) and stored in polyethene bottles (Sarstedt, 50 mL) in the fridge until analysis. Water samples for rRNA analyses (160 mL; n = 5) were collected and filtered through sterile 0.22 µm filters (Sterivex for sterile aqueous solutions). All water samples were collected directly in their respective bottle from the dripping water, with care taken to avoid contamination from the biofilms. The YBS biofilm and the bubble biofilm were sampled in 50 mL Falcon tubes, using sterile plastic spatulas. All samples for DNA extraction, amplification and sequencing were kept frozen at −80°C until DNA was extracted.

### Water analysis

Elements were analyzed by Inductively Coupled Plasma Quadrupole Mass Spectroscopy (ICP-QMS; Agilent 7500cx) using diluted external calibration solutions (Merck, Multielement Standard solution 6) and Rh as an internal mass standard. Isotopes prone to suffer from di- and poly-atomic interferences, ^39^K,  ^51^V,  ^53^Cr;  ^56^Fe, ^63^Cu, ^75^As and ^82^Se, were analyzed in collision mode using helium as the collision gas. The helium flow rate was set to 5 mL/min.

Anions were determined by ion chromatography (Metrohm) using an AG12A guard column in front of an AS12A (4 × 200 mm) separation column. External calibration was conducted using mixed standards prepared from 1 g/L single ion standards from sodium salts. Regular analysis of the ‘Fluka 89 886 certified multi standard solution’ was conducted to ensure accuracy. The 2.7 mM sodium carbonate/0.3 mM sodium bicarbonate eluent was used at a flow rate of 1 mL/min. Deionized water and 50–70 mM sulfuric acid was used as suppressor generation solutions. Carbonate/bicarbonate was measured by titration of total alkalinity, and dissolved organic carbon by DOC-analyzer (Shimadzu TOC-V CPH), following standard procedures using sodium carbonate/bicarbonate as IC-standards and potassium phthalate as TC-standard. Only polypropylene or polyethylene equipment were used in handling the samples to avoid contamination and sample alteration.

### DNA extraction

DNA was extracted from 0.5 g YBS biofilm samples (n = 7), 0.5 g bubble biofilm samples (n = 4) and water filters (n = 5) using DNeasy PowerLyzer PowerSoil kit (Qiagen). A total of five water samples, each consisting of 160 mL water, were pumped through Sterivex^TM^ 0.22 µm filters with a syringe (Millipore). The Sterivex^TM^ filters were separated from their glass casings using a sterilized hammer and a pair of sterilized tweezers (70% ethanol and Bunsen burner). The hammer was used to break the end of the glass casing and the tweezers to transfer the filter into new sterile 2 mL tubes, in which the DNA was extracted from the biomass collected on the filters.

### Small subunit rRNA gene amplification, sequencing and analysis

A two-step PCR protocol targeting the small subunit rRNA gene (16S rRNA gene in Bacteria and Archaea or 18S rRNA in Eukaryotes) was conducted using the Universal primer combination: 519 forward and 1391 reverse as described in Spang *et al*. (2015), using HotStarTaq (Qiagen) from 1 ng of template DNA. PCR conditions were as follows: 95°C for 15 min; 28 cycles of [94°C for 30 s; 57°C for 45 s; 72°C for 80 s]; 72°C for 7 min. The amplified PCR products were verified using agarose electrophoresis and purified with Ampure XP Beads 1:1 ratio as described in Spang *et al*. (2015). Primers encoding the Illumina adaptors (i5 or i7) and index sequences (8 bp) were used to amplify each PCR product from the first reaction using a second PCR as described in Spang *et al*. (2015), using 10 ng of purified DNA from the first amplification under the following reaction conditions: 95°C for 15 min; 10 cycles of [95°C for 20 s; 61°C for 30 s; 72°C for 30 s]; 72°C for 7 min. The amplified PCR products were verified using agarose electrophoresis and purified with Ampure XP Beads 1:1 ratio as described in Spang *et al*. (2015), and DNA concentration measured with Qubit High-Sensitivy assay (Thermo). Samples were normalized, pooled and submitted to the SciLifeLab sequencing facility at Uppsala University, Sweden, where they were sequenced on the MiSeq Illumina platform using Reagent kit v3, (600-cycle). A total of 16 samples were analyzed. Chimera removal, sequencing error correction and postive filtering plug-ins of QIIME v2018.11, implemented using deblur, were employed due to the observation of multiple off-target amplifications. For this, only the forward reads were analyzed. Amplicons with less than 60% sequence identity to the 88% GreenGenes database (Greengenes 13_8) were removed (Bolyen *et al*. 2018). Any sequences with only 10 reads or less across all samples were removed. Sequence features (herein described as representative operational taxonomic units, OTUs) were clustered at the 97% sequence identity level using QIIME2 (vsearch cluster-features-de-novo option). Taxonomy was assigned using a naïve Bayesian classifier in QIIME2 (feature-classifier classify-sklearn) and a confidence score of 0.7 (–p-confidence) against the SILVA v132 database.

Sequences were compared using the SILVA database (Pruesse 2007) and the NCBI BLAST network service. Sequences were deposited in the NCBI GenBank database under accession numbers SAMN11898193 to SAMN11898242 (NCBI GenBank 2019).

### Statistical analyses

Core alpha and beta diversity metrics were calculated for each comparison (i.e. all natural samples) using QIIME2 (diversity core-metrics-phylogenetic command) with representative sequences in the samples and the maximum sampling depth for retention of all samples (–p-max-depth 1100). Principal coordinate analysis (PCoA) plots were estimated using Emperor for UniFrac metrics (Lozupon *et al*. 2006). Alpha diversity (richness and evenness within samples) was assessed computing the total number of OTUs, abundance-based coverage estimator (ACE) (Chao and Lee 1992), Faith's phylogenetic diversity (Faith 1992), Shannon diversity (Shannon 1948), and Pielou's evenness (Pielou 1966) indices for all 16 samples. Differential abundance of microbial phylotypes within each subsystem was conducted through pairwise differential abundance testing using the gneiss method in QIIME2 (Morton *et al*. 2017). This approach uses a balance tree built on ratios of groups of species to infer niche differences in microbial subpopulations. The method allows exploration of relationships between proportions rather than changes in proportions of individual species (Morton *et al*. 2017). Analyses were conducted on an OTU level. Each OTU was assigned the maximum identified depth of taxonomy.

## RESULTS

### Clustering of microorganisms by subsystem

**Figure 2.**
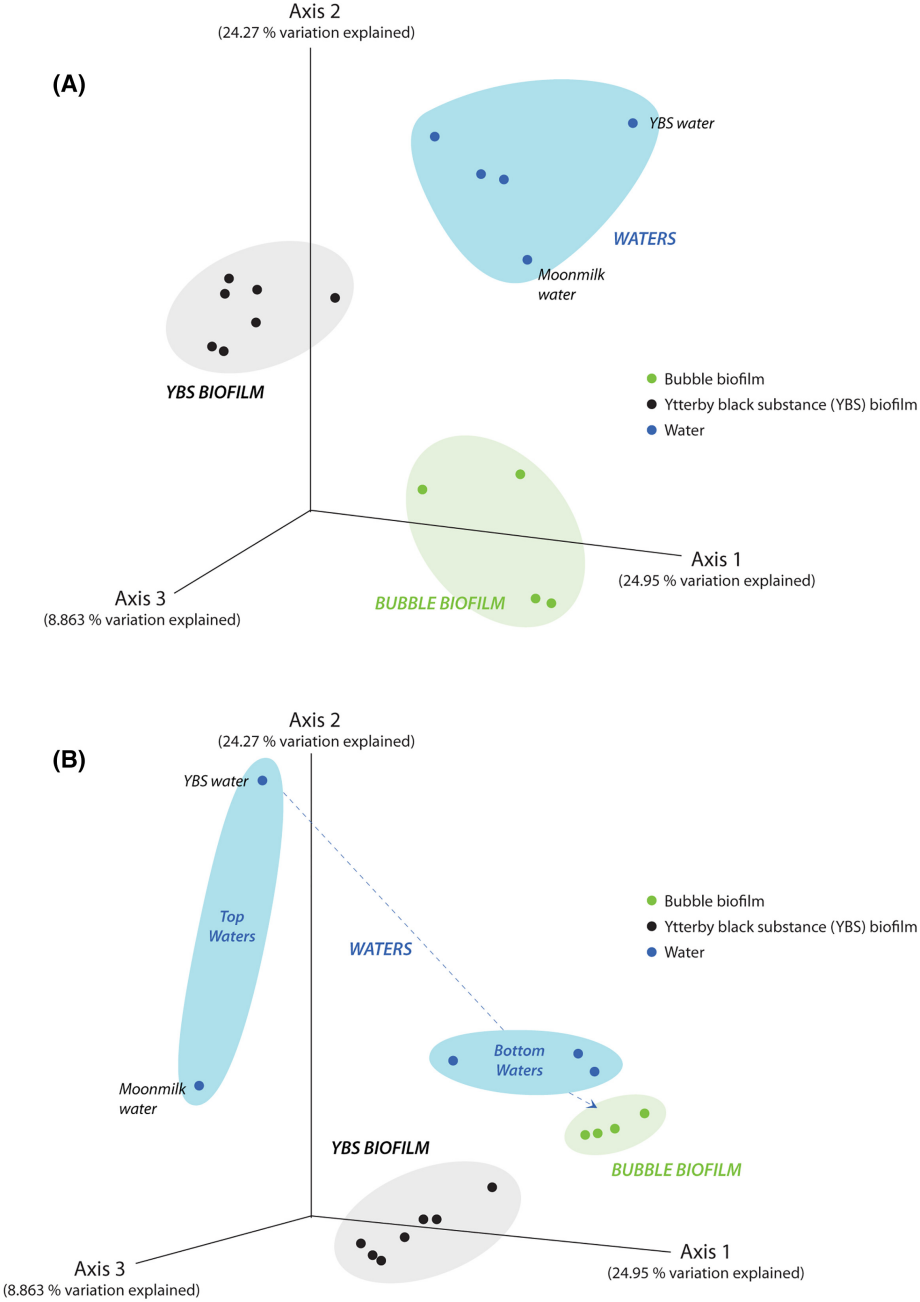
Principal coordinates plots of beta diversity (between sample types). Each data point represents a sample taken from one of the three subsystems: bubble biofilm, YBS biofilm and waters. **(A)**, Principal coordinates plot based on unweighted UniFrac distances showing variation among samples and that clustering is by subsystem type. **(B)**, Principal coordinates plot based on weighted UniFrac distances showing that feed water of the two deposits, i.e. Mn deposit water and reference moonmilk deposit water, are well separated both from each other but also from the bubble biofilm and the YBS biofilm. The bottom water samples on the other hand represent water that has percolated through the whole system and thus clusters more closely with either one of the biofilm samples, depending on the degree of interaction.

PCoA plots, used to investigate beta diversity (diversity between samples), demonstrated clustering of microorganisms primarily by subsystem (Fig. 2). There was little variation among the different YBS biofilm samples. The bubble biofilm cluster was more distinct when relative taxonomic abundance was taken into account (weighted UniFrac) compared to the analysis, which only considered the absence or presence of taxa (unweighted UniFrac). The top water samples, i.e. feed water of the Mn-oxide producing ecosystem (FW_YBS_T) and feed water of the moonmilk deposit (FW_MM_T), were well separated from each other and also from both biofilms. This was particularly visible when relative abundance of taxa was taken into account. Permutational multivariate analysis of variance (PERMANOVA) confirmed that subsystem type explained the differences in microbial population composition (*P* < 0.001).

**Figure 3.**
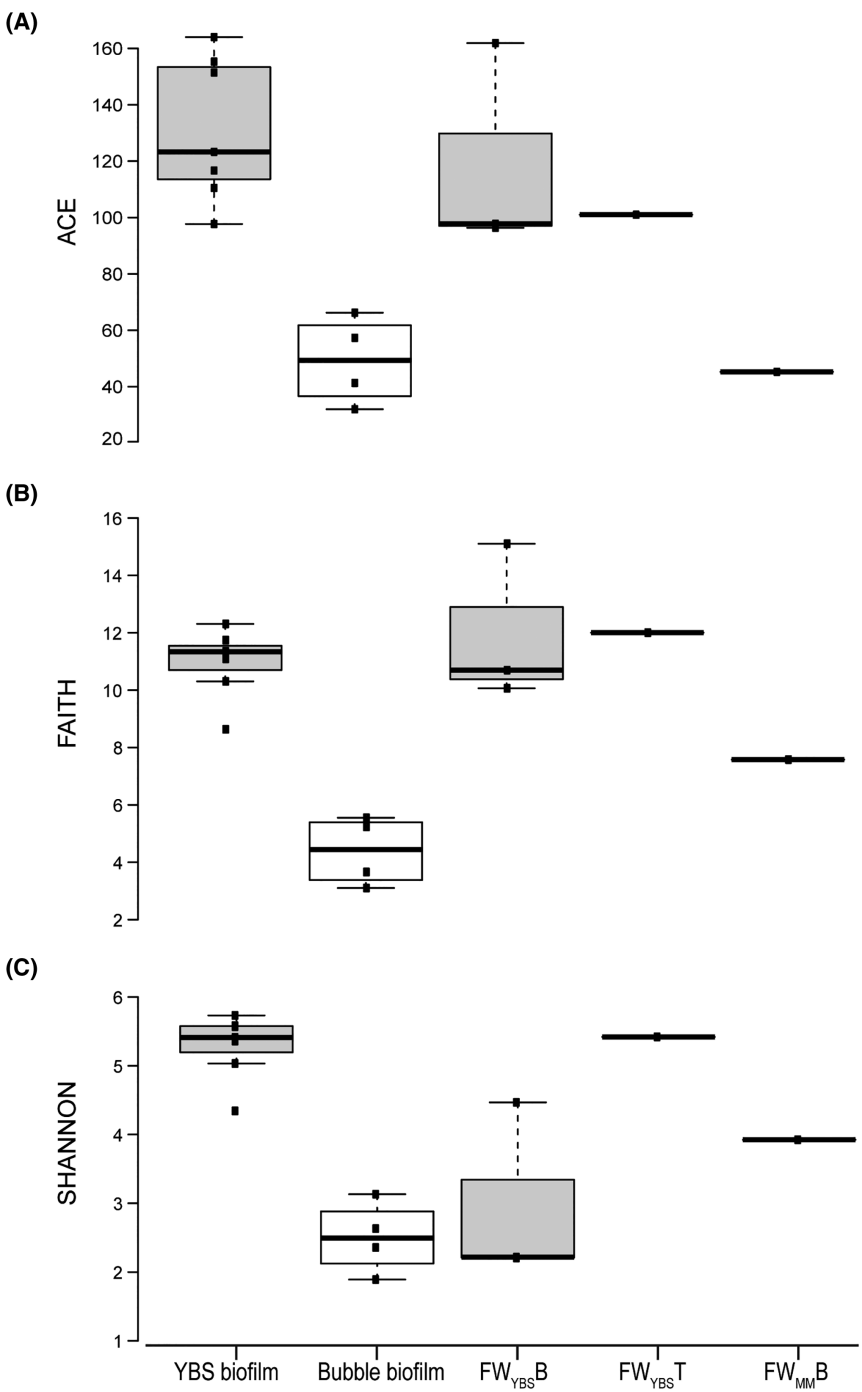
Alpha diversity boxplots showing the **(A)**, estimated richness calculated using an abundance-based coverage estimate (ACE), **(B)**, Faith's phylogenetic diversity and **(C)**, Shannon diversity of microbiomes in the Ytterby mine tunnel. The two top fracture water samples (FW_YBS_T and FW_MM_T) are depicted individually in order to visualize the large variations between these two water samples. The horizontal lines inside the boxes represent the median values, and the lower and upper ends of the boxes represent the first and third quartiles, respectively. Whiskers represent sample values outside the 50% mid-range. Squares outside the whiskers represent sample values > 1.5 times the height of the box.

The alpha diversity (within samples distances) was assessed using a combination of indices (SI.1) and a representative selection of these measurements are shown in Fig. 3. The YBS biofilm, with the exception of one bottom water sample, was the most species rich subsystem (samples ranging from 90 to 138 observed OTUs and 98–169 expected total number of OTUs calculated using the abundance-based coverage estimator, ACE). The FW_YBS_T was in the same range (94 observed OTUs and 101 expected ones) as the YBS biofilm but substantially higher than the bubble biofilm (samples ranging from 26 to 47 observed OTUs and 32–66 expected ones) (Fig. 3A). The microbial population in the bubble biofilm was also the least phylogenetically diverse (Faith indices ranging from 3.1 to 5.5) while the FW_YBS_T (Faith index 12) including one of the samples in the FW_YBS_B group (Faith index 15.1) and the YBS biofilm (Faith indices ranging from 8.6 to 12.3) were the most diverse (Fig. 3B) (Faith 1992). Surprisingly both species richness and phylogenetic diversity in the reference moonmilk water (FW_MM_T) were considerably lower than those of the FW_YBS_T. The YBS biofilm and the FW_YBS_T (5.4) had the highest Shannon diversity (ranging from 4.3 to 5.7) indicating a relatively even distribution of species abundance among the OTUs. The comparatively low Shannon measure in the bubble biofilm (ranging from 1.9 to 3.1) reflects the dominance of a small number of OTUs in these samples (Fig. 3C). These conditions are also represented by the Pielou's eveness indices where a value of 1 is representative of each species being equally likely. Kruskal-Wallis pairwise comparisons between the three subsystems showed that all indices were significantly different (*P* < 0.05) between the YBS biofilm and the bubble biofilm but that waters only differed significantly from bubble biofilm in terms of phylogenetic diversity. Despite the fact that water samples formed a cluster well separated from the two biofilm types in the unweighted PCoA, all measures that involved relative species abundance showed large variation within the group. FW_YBS_B percolated through both biofilms on its path down the rock wall and the large variations observed between these samples likely reflect variations in water-biofilm interference. As a result these water samples are unpredictable mixtures of all subsystems in the Mn-oxide producing ecosystem.

### Microbial community composition in fracture water

**Figure 4.**
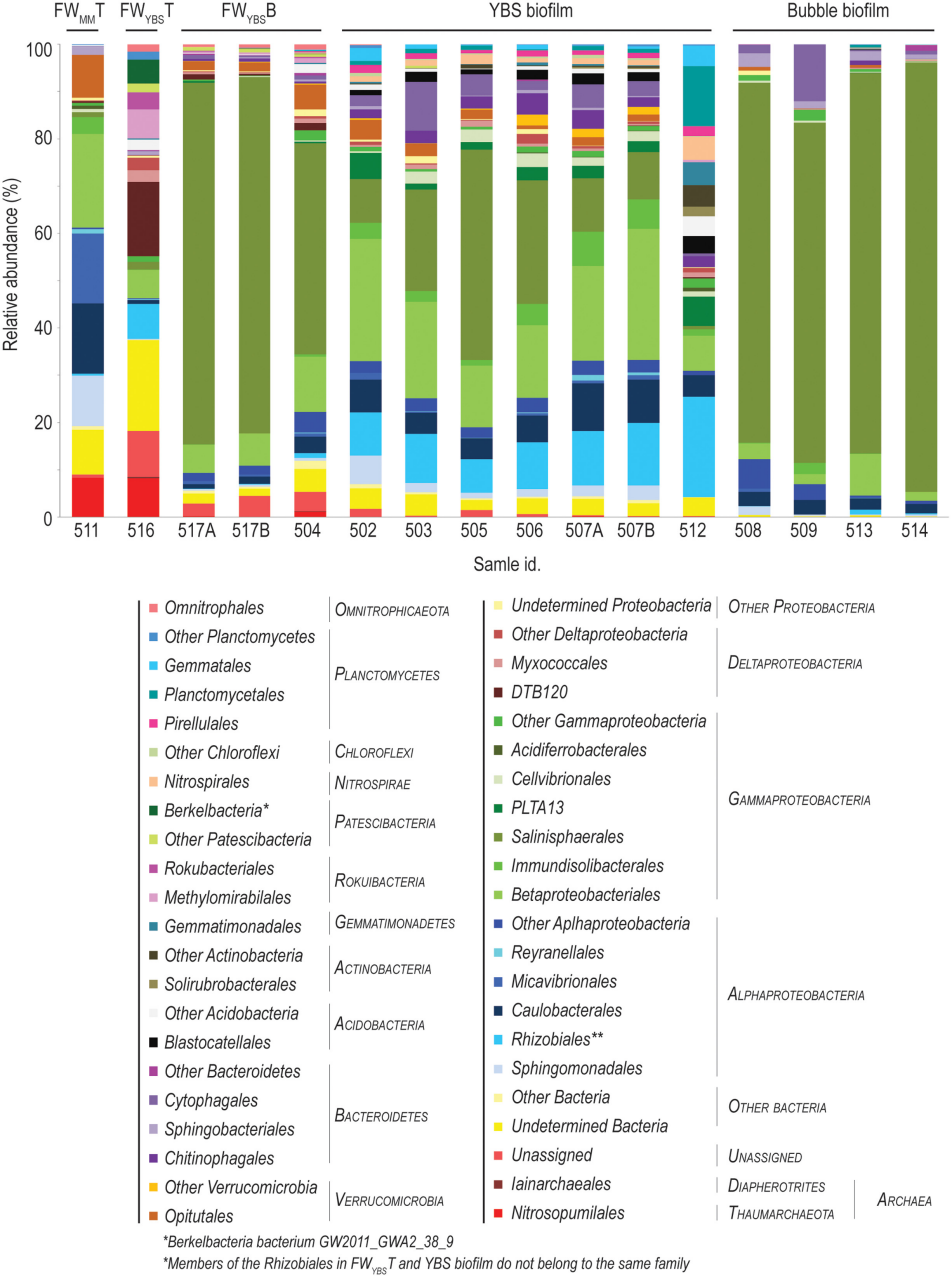
Microbial community composition based on 16S rRNA gene analyses. Taxonomic assignment is made at order level. Labels on the horizontal axis indicate sample type (above the chart) and individual sample identity (below the chart). Sample types are divided into fracture feed water of the moonmilk deposit (FW_MM_T), fracture feed water of the Mn-oxide producing ecosystem FW_YBS_T), fracture water that has passed through the biofilms down the approximately 2 m tall rock wall (FW_YBS_B), Mn-oxides and associated biofilm (YBS biofilm) and biofilm forming bubbles on the Mn-oxides (bubble biofilm).

The high relative abundance of archaea in the top waters stood out from the other samples with 8% 16S rRNA gene reads in each top water sample compared to maximum 0.7% in the other subsystems (Fig. 4). Members of the *Nitrosopumilaceae* family within the *Thaumarchaeota* dominated these archaeal communities and were mainly associated with an OTU most similar to an uncultured archaeon clone from subsurface Hanford site (HM187506, Lin *et al*. 2012) which is contaminated by a number of pollutants, i.e. metals, radionuclides and organic solvents. In addition, the top water also had a high proportion of unassigned sequences (9.7% 16S rRNA gene reads) that all were most similar to uncultivated environmental archaea. The most abundant of these OTUs represented 6.1% and was most similar (94%) to an archaean gene sequence from a study of deep repositories for liquid radioactive waste (GQ221437, Nazina *et al*. 2010).

The feed water of the Mn-oxide producing ecosystem (FW_YBS_T) had a high proportion of *Deltaproteobacteria*, with 20.8% 16S rRNA gene reads compared to 0.5% in the moonmilk water (Fig. 4). The most predominant phylotype was the environmental group DTB-120 that is associated with Fe-cycling in deep sea hydrothermal environments (Makita *et al*. 2016). Also members of the microaerophilic Fe(II)-oxidizing family *Gallionellaceae* (Hallberg and Broman 2018) were present in the fracture water (2.5%), but virtually absent in all the other natural samples, including the moonmilk water (FW_MM_T).

Another abundant group in the FW_YBS_T was *Rokubacteria* belonging to the candidate division NC10, with 9.7% 16S rRNA gene reads compared to non presence in the FW_MM_T. Sequences within this group were associated with the methane oxidizing bacterium *Candidatus Methylomirabilis*, with 6.1% 16S rRNA gene reads (pathway that involves anaerobic production of oxygen used for methane oxidation) and uncultured members of the *Rokubacteriales* (3.6%). Members of the *Patescibacteria* superphylum represented a high proportion of the microbial community in the FW_YBS_T (6.9% 16S rRNA gene reads) while absent in the two biofilms and the FW_MM_T. Sequences were mainly associated with two OTUs, *Berkelbacteria bacterium* GW2011_GWA2_38_9 (KX123472, Brown *et al*. 2015) and *Parcubacteria* GW2011_GWB1_41_6, (KX123488, Brown *et al*. 2015). The absence observed in the biofilms is in accordance with a previous study in the deep subsurface where this taxon (*Parcubacteria*) was observed almost exclusively as planktonic cells (Wu *et al*. 2017). The top water samples were also the two samples with the highest relative abundance of unclassified bacteria: 19.2% of the FW_YBS_T and 9.4% of the FW_MM_T. Corresponding numbers for the bubble biofilm and YBS biofilm were approximately 0.5% and 4%, respectively.

*Alphaproteobacteria* constituted a substantially larger part of the microbial population in FW_MM_T (42% 16S rRNA gene reads) compared to the FW_YBS_T (8.8%) (Fig. 4). In addition, the two systems hosted divergent taxa within the *Alphaproteobacteria*. The majority of retrieved sequences from the FW_YBS_T belonged to the *Mesorhizobium* genus within the *Rhizobiaceae* family (7.4% 16S rRNA gene reads) whereas the FW_MM_T mainly consisted of members of an uncultured genus within the *Micavibrionaceae* family (14.8% 16S rRNA gene reads), the *Brevundimonas* genus within the *Caulobacteraceae* (14.8%) and *Sphingobium* within the *Sphingomonadaceae* (10.6%). Sequences belonging to the *Mesorhizobium* genus in the FW_YBS_T were grouped into one OTU (172) which was 98.5% similar to *M. Australicum* TG-1, an acid tolerant Mn-oxidizing bacterium (HG932494, Bohu *et al*. 2015). A high relative abundance of the *Opitutaceae* family within the *Verrucomicrobia phylum* (9.0%) were observed in the FW_MM_T whereas this group of bacteria was close to absent in the FW_YBS_T (0.1%).

### Differentially abundant taxa in the YBS biofilm

**Figure 5.**
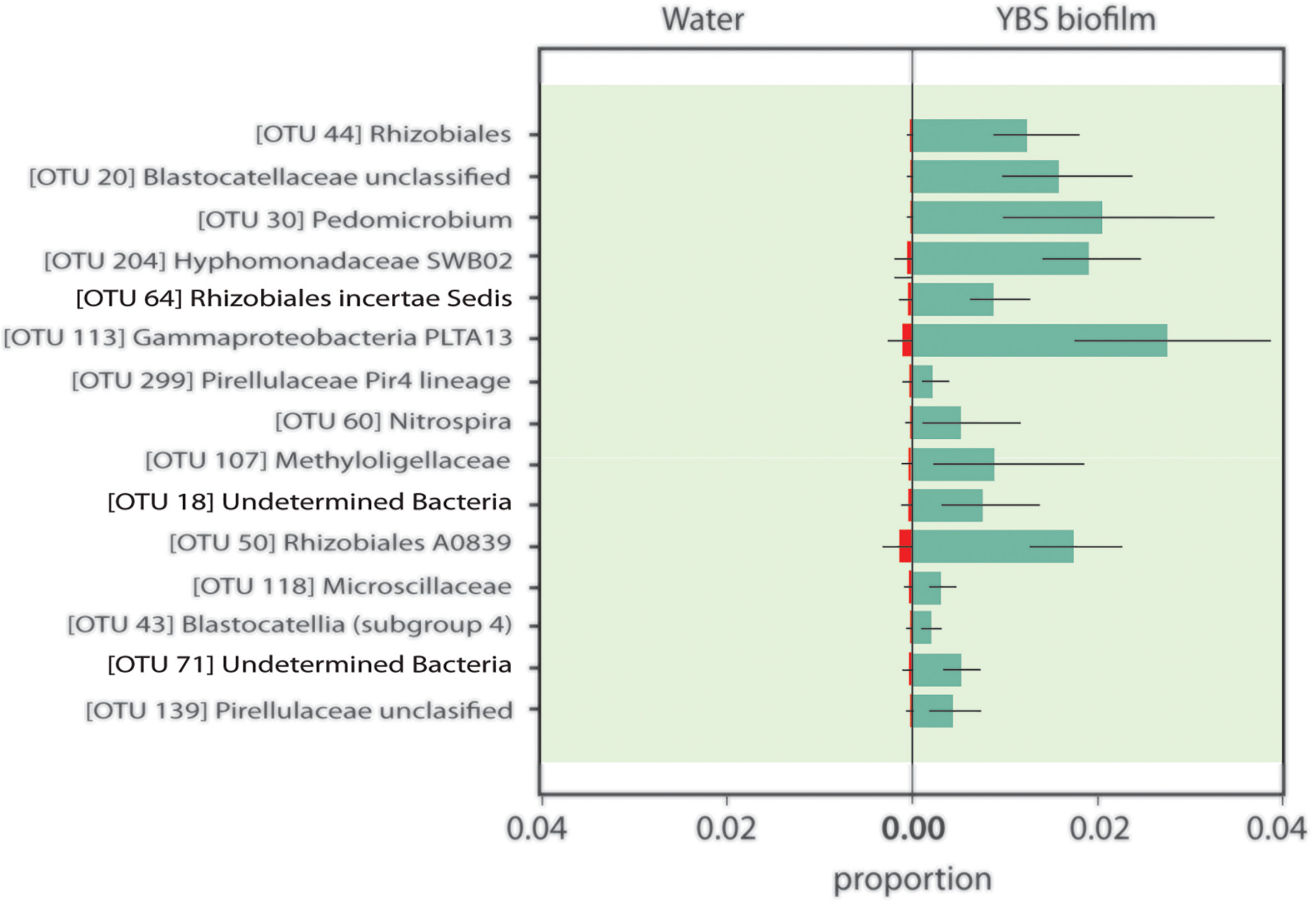
Proportion plot showing differentially abundant taxa in the YBS biofilm. The plot was constructed through pairwise differential abundance testing using the gneiss method in QIIME2 (Morton *et al*. 2017). This approach uses log-transformed ratios of groups of species to infer niche differences in microbial subpopulations. Analyses were conducted on an operational taxonomic unit (OTU) level. Each OTU was assigned the maximum identified depth of taxonomy. Proportions correspond to mean values and standard deviations based on all samples within the YBS biofilm sample group.

Differentially abundant bacteria in the YBS biofilm were identified within the *Rhizobiales*, PLTA13 *Gammaproteobacteria, Pirellulaceae, Hyphomonadaceae, Blastocatellaceae* and *Nitrospira* (Fig. 5). Within the *Rhizobiales* order *(Alphaproteobacteria)* five differentially abundant OTUs were identified. Surprisingly, none of these OTUs were observed in the feed water (FW_YBS_T), but constituted 4.6 to 12.6% of the relative abundance within the YBS biofilm samples. The most abundant of these *Rhizobiales* (OTU 30) was affiliated to the hyphae budding, ferromanganese genus *Pedomicrobium* (Gebers 1981). Another one (OTU 107) was assigned to the methylotrophic *Methyloligellaceae* family and the other three OTUs were not possible to assign a deeper taxonomic affiliation than the order *Rhizobiales*. The differentially abundant sequences within the *Hyphomonadaceae* clustered into one OTU (204), 99% similar to an isolated Mn-oxidizing strain (LC270264, Okano *et al*. 2016) and the uncultivated environmental group PLTA13 *Gammaproteobacteria* (OTU 113) was identical to a sequence retrieved from a hot spring Mn deposit (LC422457, Shiraishi *et al*. 2019). Members of the *Pirellulaceae* family (*Planctomycetes*) grouped into two OTUs (139 and 299) and the *Blastocatellaceae* sequences (*Acidobacteria* phylum) mainly into one OTU (20), 96.3% similar to *Stenotrophobacter terrae* strain Ac_28_D10 (NR_146 023, Pascual *et al*. 2015). The differentially abundant bacteria within the *Nitrospira* genus grouped into one OTU (60). A recent study established that a bacterium affiliated to the phylum *Nitrospirae* was capable of Mn(II)-oxidation coupled to energy conservation (chemolithoautotrophy), a central question within the Mn field that has long been investigated (Yu and Leadbetter 2020).

Members of the *Chitinophagaceae* and *Saprospiraceae* families within the *Chitinophagales* order in *Bacteroidetes* composed a higher relative abundance (but not significantly differentially abundant) of the YBS biofilm samples compared to the waters and bubble biofilm (1.9 to 4.6% 16S rRNA gene reads compared to < 0.9% in the other sample types, Fig. 4). Part of the *Bacteroidetes* cluster identified in our previous study (Sjöberg *et al*. 2018) was detected in the *Chitinophagaceae* family (Ytterby clone 1 A02, MG657061). Within the *Saprospiraceae* family, the genus *Haliscomenobacter* was represented together with groups of undetermined *Saprospiraceae* most similar to clones from lithifying microbialites (KP589290 and KP514320, Corman *et al*. 2016), acid mine drainage sediments (KY943160, Ramanathan *et al*. 2017), and a study on Mn-oxidizing bacteria during biofilm formation (AF379685, Kielemoes *et al*. 2002). The core microbial groups in YBS biofilm sample 512, collected from the same area as the other YBS biofilm samples three years earlier, are the same but the older sample differs by a higher relative abundance of *Planctomycetales*.

### Differentially abundant taxa in the Bubble biofilm

Bubble biofilm and bottom water samples had the highest concentration of *Gammaproteobacteria* of all samples (biofilm between 79% and 93% of the total prokaryotic community and bottom waters between 59% and 83%). These samples also had a similar *Gammaproteobacteria* profile clearly dominated by sequences belonging to *Salinisphaerales* order and in particular the *Nevskia* genus (Fig. 4) (Sjöberg *et al*. 2020). This group of strictly cheomorganotrophic aerobes are mainly found in shallow water environments where they form floating biofilms at the air-water interface, defined as epineuston (Stürmeyer *et al*. 1998). These bacteria were clustered into two OTUs (17 and 36). Only one of these OTUs (17) was significantly differentially abundant for the bubble biofilm while the other OTU (36) was common in all sample types with the exception of the top waters where *Nevskia* only constituted maximum 0.9% in total. The bubble biofilm specific OTU was 97.76% similar to *Nevskia ramosa* strain Soe1 DSM 11499 (NR_025269, Stürmeyer *et al*. 1998) while the other group of *Nevskia* was 98.9% similar to *Nevskia ramosa* strain MAFF 211643 (AB518684, Kawai, NCBI GenBank 2019).

### Shared bacterial taxa

Members of the *Rhodocyclaceae* family within the *Betaproteobacteriales* constituted a substantial part of the microbial population in all subsystems, but most so in the YBS biofilm and the FW_MM_T (up to 24% of the 16S rRNA gene reads) (Fig. 4). Sequences were grouped into one single OTU and identified as *Sulfuritalea* using the QIIME2 feature-classifier naïve Bayesian method. However, the NCBI database showed that this OTU was even more similar (99%) to *Rugosibacter aromaticivorans* strain Ca6 (NR_156 019), isolated from contaminated soil and capable of degrading aromatic compounds (Corteselli, Aitken and Singleton 2017a). *Sulfuritalea* is frequently observed at hydrocarbon contaminated sites and closely related to aromatic compound degrading bacteria (Sperfeld, Diekert and Studenik 2019). Furthermore, the *Immundisolibacter* genus within the *Gammaproteobacteria* constituted an important proportion of the microbial community in the YBS samples (1.1% to 7.3% of the 16S rRNA gene reads) (Fig. 4). Sequences were most similar to *Immundisolibacter cernigliae* strain TR3.2 (NR_156 801), a polycyclic aromatic hydrocarbon degrading bacterium (Singleton *et al*. 2016; Corteselli, Aitken and Singleton 2017b). This group of bacteria was also present in the FW_MM_T (3.6% of the 16S rRNA gene reads) but virtually absent in the FW_YBS_T (0.1%).

### Occurrence of potential Mn-oxidizing microorganisms

Potential Mn(II)-oxidizers are mainly detected in the YBS biofilm, but also as one group *(Mesorhizobium*) almost unique for the FW_YBS_T (Table 1). In addition, it appears that one of the highly abundant taxa observed in all subsystems (*Sulfuritalea*) is involved in Mn-cycling but it is unclear whether this bacterial group oxidize soluble Mn or if it uses Mn(III/IV) as an electron acceptor. Two Mn-oxidizing bacterial species (*Hydrogenophaga* sp., *Pedobacter* sp.) and one fungus (*Cladosporium* sp.) were also previously isolated from the Ytterby system, grown in the dark at a low temperature of 8°C (Sjöberg 2019). The two bacterial species accounted for a maximum of 0.09% and 0.03% respectively in the YBS biofilm sequence data, while the fungus was not detected at all. One group of unclassified *Burkholderiaceae* was 94.7% similar to the isolated *Hydrogenophaga* sp. (average relative abundance in YBS biofilm samples 1.4%). Yet another bacterial species (*Rhizobium* sp.) was observed to be involved in Mn-oxidation but results implied a synergistic relationship with other species (Sjöberg 2019).

**Table 1.** Potential Mn-oxidizers (% relative abundance, average for sample type) detected in fracture feed water of the moonmilk deposit (FW_MM_T), in fracture feed water of the Mn-oxide producing ecosystem (FW_YBS_T), in fracture water that has passed through the biofilms down the approximately 2 m tall rock wall (FW_YBS_B), in Mn-oxides and associated biofilm (YBS biofilm), in biofilm forming bubbles on the Mn-oxides (bubble biofilm).

	% total seqences (average for sample type)					
Taxonomic category (phylum—genus)	FW_MM_T	FW_YBS_T	FW_YBS_B	YBS biofilm	Bubble Biofilm	Reference to related Mn-oxidizers*
***Bacteria***						
***Alphaproteobacteria***						
Pedomicrobium	-	-	0.02	2.90	0.07	Ghiorse and Hirsh 1979; Gebers 1981
Hyphomicrobium	-	0.11	0.09	1.30	0.02	Ghiorse 1984; Bohu *et al*. 2015
Hyphomonadaceae SWB02 (SILVA)	-	-	0.10	2.00	0.05	Okano *et al*. 2016 (99% similar to isolated Mn-oxidizer, NCBI accession no. LC270264)
Methyloligellaceae unclassified	-	-	0.04	0.93	0.03	Molari *et al*. 2020
Rhizobiales unclassified	0.43	0.17	0.17	5.41	0.19	Northup *et al*. 2010; Bohu *et al*. 2015 (Rhizobium)
Mesorhizobium	-	7.10	0.07	0.07	-	Bohu *et al*. 2015
Reyranella	0.94	0.14	0.04	0.09	-	Marcus *et al*. 2017
***Gammaproteobacteria***						
PLTA13 env. Group (SILVA)	-	-	0.23	3.20	0.04	Shiraishi *et al*. 2019 (identical to sequence retrieved from Mn crust, NCBI accession no. LC422457)
Hydrogenophaga sp.**	-	0.03	0.02	0.03	0.04	Marcus *et al*. 2017; Sjöberg 2019
Sulfuritalea**	17.4	1.3	8.27	13.8	3.58	Breda *et al*. 2017
***Bacteroidetes***						
Cythophaga	-	-	0.10	-	-	Northup *et al*. 2010; Carmichael and Bräuer 2015
Terrimonas	-	0.10	-	0.80	0.1	Northup *et al*. 2010; Carmichael and Bräuer 2015
Pedobacter sp.**	-	-	-	0.01	0.01	Sjöberg 2019
Planctomycetes						
Pirellulaceae unclassified	-	-	0.04	0.93	-	Molari *et al*. 2020
Pirellulaceae Pir4 lineage	-	-	0.03	0.35	-	Molari *et al*. 2020
***Actinobacteria***						
Pseudonocardia	-	-	-	0.20	-	Cahyani *et al*. 2009; Carmichael and Bräuer 2015
***Acidobacteria***						
Blastocatellia subgroup 4	-	-	-	0.21	-	Molari *et al*. 2020 (Blastocatella)
Blastocatellaceae unclassified	-	-	-	1.83	0.04	Molari *et al*. 2020 (Blastocatella)
Blastocatella	-	-	-	0.20	-	Molari *et al*. 2020
***Nitrospirae***						
Nitrospira	-	-	0.12	1.80	0.06	Yu and Leadbetter 2020 (Nitrospirae)
***Fungi***						
***Ascomycota***						
Cladosporium sp.**	-	-	-	-	-	Santelli *et al*. 2011; 2016; Sjöberg 2019

*References refer either to isolated Mn-oxidizing bacteria/fungi or to bacteria associated with Mn-oxide rich environments.

** Members of the *Sulfuritalea* genus have been detected in polystyrene filters used for Mn-removal in drinking water systems. It appears that these bacteria are involved in Mn-cycling but it is unclear whether they oxidize soluble Mn or use Mn(III/IV) as an electron acceptor

The most represented group within the differentially abundant *Rhizobiales* was affiliated to the gram negative, hyphae budding, ferromanganese genus *Pedomicrobium* which is a known Mn-oxidizer and frequently observed in underground settings and as part of the microbial community composition in ferromanganese nodules (Gebers 1981; Giorse 1984; Northup *et al*. 2003). Mn-oxidation by *Pedomicrobium* spp. is a two-step mechanism, in which negatively charged extracellular organic matter, produced by the cells, attract soluble Mn which thereafter is oxidized and deposited on the surface of these bacteria (Ghiorse and Hirsch 1979). Three of the differentially abundant OTUs in the YBS biofilm (*Methyloligellaceae* unclassified and *Pirellulaceae* unclassified and *Pirellulaceae* Pir4 lineage) were found to be more abundant in deep-sea Mn nodules relative to surrounding sediments (Molari *et al*. 2020). An interesting group is the *Gammaproteobacteri*a PLTA13 (SILVA) cluster which was differentially abundant in the YBS biofilm. This cluster was identical to a sequence retrieved from a hot spring Mn deposit (LC422457, Shiraishi *et al*. 2019) but only 91.42% similar to the closest cultivated relative, a purple sulfur bacterium, *Ectothiorhodospira mobilis* strain DSM 237 (NR_125 567, Swiderski, J., NCBI GenBank 2020).

### Water chemistry and YBS composition

Elemental concentrations in fracture water sampled at the top of the rock wall where water emerges from the fracture (FW_YBS_T), and water sampled at the bottom of the rock wall after it has passed through the biofilms and thus the Mn precipitation zone (FW_YBS_B) are summarized in (Table 2). Composition of the fracture water at the top of the MM precipitation zone (FW_MM_T), located ca 50 m from theMn-oxidizing system, is also given. Elemental data for the YBS biofilm are averages from four measurements (Sjöberg *et al*. 2017).

**Table 2.** Concentrations of elements (above 10 mg/kg, besides C, O and Si) in the YBS and the moonmilk (MM) precipitates, composition of the fracture water on top (FW_YBS_T) and bottom (FW_YBS_B) of the YBS precipitation zone, as well as fracture water on top of the MM precipitation zone (FW_MM_T). Concentrations in solids are given in mg/kg and concentrations in water (filtered samples, 0.2 µm filters) are given in µg/L. Levels of dissolved organic carbon (DOC) are in the range 4–6 mg/L (Allard *et al*. 2019). Concentrations of total carbon (TC), total organic carbon (TOC) and total nitrogen (TN) for the YBS are 1.81%, 0.59% and <0.1%,  respectively (Sjöberg *et al*. 2017).The concentration ratio between filtered and non-filtered samples (%) is given within parenthesis for Mn and Fe. Enrichment factor (k_e_) is defined as the [YBS]/[FW_YBS_T] concentration ratio (L/kg). Elemental concentrations in the bubble biofilm are not measured.

	YBS*	FW_YBS_T**	FW_YBS_B**	FW_YBS_B/ FW_YBS_T**	log k_e_	MM	FW_MM_T**
**pH**		8.28	8.33	-	-	-	8.3
**HCO_3_^−^**		205 000	228 000	1.11	-	-	240 000
**NO_3_^−^**		290	510	1.76	-	-	400
**F^−^**		330	300	0.91	-	-	300
**SO_4_^2^^−^**		29 020	35 220	1.21	-	-	38 000
**Cl^−^**		20 900	20 680	0.99	-	-	21 000
**Mn**	452 710	172 (99)	2.72 (65)	0.016	6.4	14	0.04 (4.0)
**Ca**	64 170	56 360	61 740	1.09	3	378 600	62 400
**REE**	10 095	10.81	7.03	0.65	6	-	4.01
**Mg**	4075	8340	8140	0.98	2.7	1417	7280
**Ba**	1765	4.5	4.17	0.93	5.6	21	5.36
**Cu**	1555	3.89	3.4	0.87	5.6	24	5.65
**Al**	841	5.26	5.14	0.98	5.2	344	6.31
**V**	683	2.68	1.82	0.68	5.4	1	1.87
**K**	666	1720	1690	0.98	2.6	54	1790
**Sr**	639	250	237	0.95	3.4	319	287
**Fe**	583	0.40 (68)	0.044 (63)	0.11	6.2	688	1.35 (55)
**Na**	399	24 510	24 080	0.98	1.2	290	37 800
**Zn**	276	0.75	0.65	0.87	5.6	57	1.02
**Co**	157	0.22	0.13	0.59	5.6	1	0.19
**Mo**	125	1.86	1.69	0.91	4.8	1	2.86
**Pb**	52.8	<0.1	<0.1	-	>5.7	18	0.02
**Ga**	39.5	0.13	0.11	0.85	5.5	1	0.13
**As**	37.3	0.35	0.13	0.37	5	3	0.53
**Ni**	34.3	1.04	0.99	0.95	4.5	11	1.21
**Cd**	11.8	<0.1	<0.1	-	>5.1	<1	<0.1

* Average of four YBS samples (Sjöberg *et al*. 2017).

** Waters sampled within one hour.

(-) Data non applicable or not measured.

**Figure 6.**
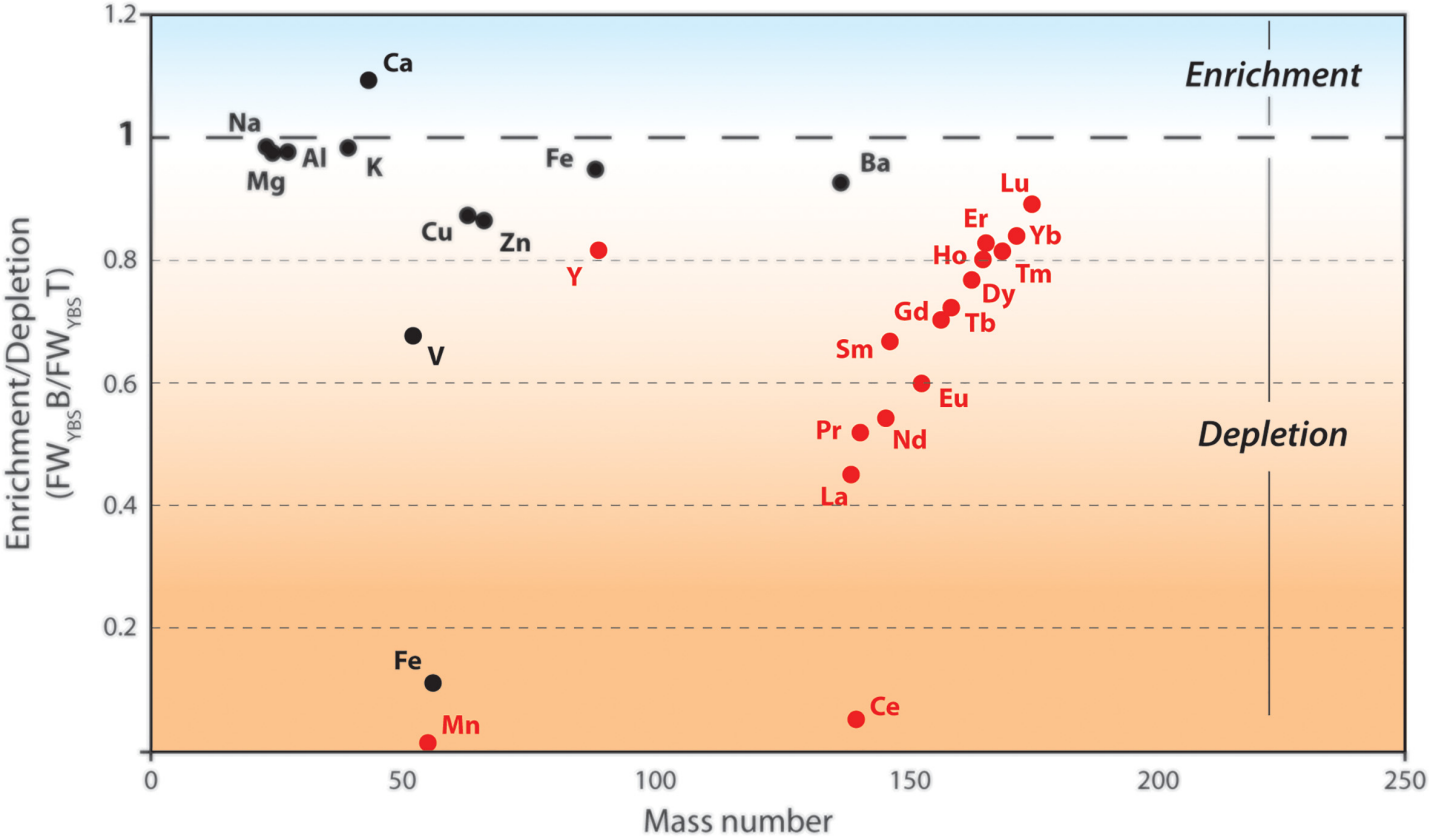
Plot showing whether an element is enriched or depleted from the fracture water after it has percolated through the Mn precipitation zone: from the top of the rock wall as the water emerges from the fracture (FW_YBS_T), to the bottom of the deposit after it has passed through the Mn precipitates down the 2 m tall rock wall (FW_YBS_B) (modified from Sjöberg 2019). Enrichment or depletion of an element is expressed as concentration ratios, FW_YBS_B/FW_YBS_T and are plotted *vs* atomic mass. The broken line corresponds to no change in elemental concentration. The plot shows a strong depletion of particularly Mn and Ce, as well as Fe and the lighter REE. The lighter the REE is (less atomic mass, larger ionic radius), the stronger is the depletion from top to bottom water (except from Ce that is more depleted than La) (Data for individual REE from Allard *et al*. 2019).

Data reveal important differences in fracture water composition above and below the Mn precipitation zone and also between fracture water feeding the two chemically and physically different deposits ( (Table 2). About 98% and 89% of dissolved Mn and Fe, respectively, are lost from the aqueous phase as the water travels through the he Mn precipitation zone down the rock wall. Fe concentrations are however very low. What stands out is the Mn concentration in the FW_YBS_T (172 µg/L) which drops to only 3 µg/L in the FW_YBS_B and is substantially higher than that of the FW_MM_T (˂1 µg/L). Also the REE concentrations in the FW_YBS_T are more than twice as much as those of the FW_MM_T (11 µg/L vs. 4 µg/L, respectively) and drops to 7 µg/L in the FW_YBS_B. Ratios of elemental concentrations (FW_YBS_B/FW_YBS_T) were plotted to illustrate whether an element becomes enriched or depleted from the fracture water as it travels through the Mn precipitation zone (Fig. 6). The plot shows a strong depletion of particularly Mn and Ce, as well as Fe and the lighter REE. As the water travels through the Mn precipitation zone, REE fractionation take place. Going from a ratio of 0.45 for La, being the lightest and largest ion in the series (radius 103 pm) to 0.89 for, Lu (radius 86 pm), being the heaviest and smallest.

All divalent trace metals, except Ca and Mg, are slightly depleted, possibly reflecting adsorption on the fresh Mn precipitates. Levels of major anions are increasing (HCO_3_^−^, SO_4_^2−^) as are calcium among the cations.

Turning to the YBS biofilm, its main component was previously identified as a Mn-oxide of the birnessite-type, with the general formula M_x_(Mn(III, IV)_2_O_4_∙(H_2_O)_n_ (Sjöberg *et al*. 2017). Here, x is around 0.4, and M is Ca, REE and Mg, possibly with minor contributions of other elements of similar ionic radius as Ca and the REE. The biofilm preferentially accumulates the trivalent REE over divalent and monovalent cations. There is also a preferential uptake of light rare earth elements (LREE) relative to heavy rare earth elements (HREE), likely as a response to mineralogical preferences (ionic radii for LREE are similar to Na and Ca which are the main charge balancing cations in the generic mineral formula for birnessite; Sjöberg *et al*. 2017). The birnessite phase constitutes ca 98.5% of the total YBS mass with respect to all elements with the exception of H, C, O and Si. The remaining 1.5% corresponds to some 15 elements at concentrations above 10 mg/kg (Table 2 ) and 10 more elements at levels between 1 and 10 mg/kg (not given in Table 2). These elements are adsorbed or co-precipitated, or to a minor extent included in the birnessite structure in inner-sphere positions (Allard *et al*. 2019).

## DISCUSSION

### Fracture water chemistry and diversity—implications for biofilm formation

The metal enriched, low carbon YBS biofilm, growing in the dark at low temperature, forms a seemingly harsh microbial habitat. Although the chemistry of the fracture feed water does not reflect this extreme character, it exerts substantial control on biofilm formation. A comparison between FW_YBS_T and the adjacent FW_MM_T shows that important differences exist. Both fracture waters are similar with respect to major components with a Ca-HCO_3_–Na-Cl-SO_4_ signature and pH around 8.3. They are both close to saturation with respect to CaCO_3_, assuming a solubility product for CaCO_3_(s) of 3.3 × 10^−9^ (Aylward and Findlay 2002). Dissolved organic carbon (DOC) is of the order 4–6 mg/L which is in the lower range of shallow groundwater from the region (Augustsson, Bergbäck and Åström 2009). Also concentrations of trace components, down to the 1 μg/L-level, are similar. What stands out is the Mn concentration in the FW_YBS_T (172 µg/L) which is substantially higher than that of the FW_MM_T (˂1 µg/L). A comparable shallow groundwater from the region ranges from 20–150 µg/L, 10th and 90th percentiles (Augustsson, Bergbäck and Åström 2009). Also the REE concentrations in the FW_YBS_T are more than twice as much as those of the FW_MM_T (11 µg/L vs. 4 µg/L, respectively) and in the upper range, but not higher, of regional values (Mathurin *et al*. 2014). So, despite similarities in water chemistry, the two adjacent water-bearing fractures carry water that differs substantially in its Mn and REE concentrations.

Alpha diversity metrics reveal large variations between the planktonic populations in these two water-sources, both in terms of species richness and phylogenetic diversity, (SI. 1; Fig. 3). This implies that feed water diversity largely is determined by bedrock and fracture characteristics. In contrast, the community composition in water sampled at the bottom of the rock wall, after it has passed through the Mn precipitates (FW_YBS_B) is an unpredictable mixture of all of the above subsystems (feed water and biofilms). Data show that certain microbial groups follow the water all the way across the system without attaching to either one of the biofilms. They simply flush through the system.

However, both the YBS and bubble biofilm each have very specific communities which to a large extent are independent of the planktonic community. The differentially abundant taxa in the YBS biofilm are not even detected in the FW_YBS_T but rather emerge in the YBS biofilm. This implies that the dominant microbial groups in the feed water have little influence on the derived biofilms. Rather, it seems that the divergent community compositions in the biofilms result from a process of selection, concentration and enrichment of prokaryotic groups that take advantage of the new conditions offered. It is possible that a highly diverse, species-rich planktonic community have a greater chance of hosting an organism that could take advantage or adjust to specific conditions, potentially leading to the emergence of a new niche. This seems to be the case with the YBS and bubble biofilms.

### Exchange reactions between biofilm and fracture water

**Figure 7.**
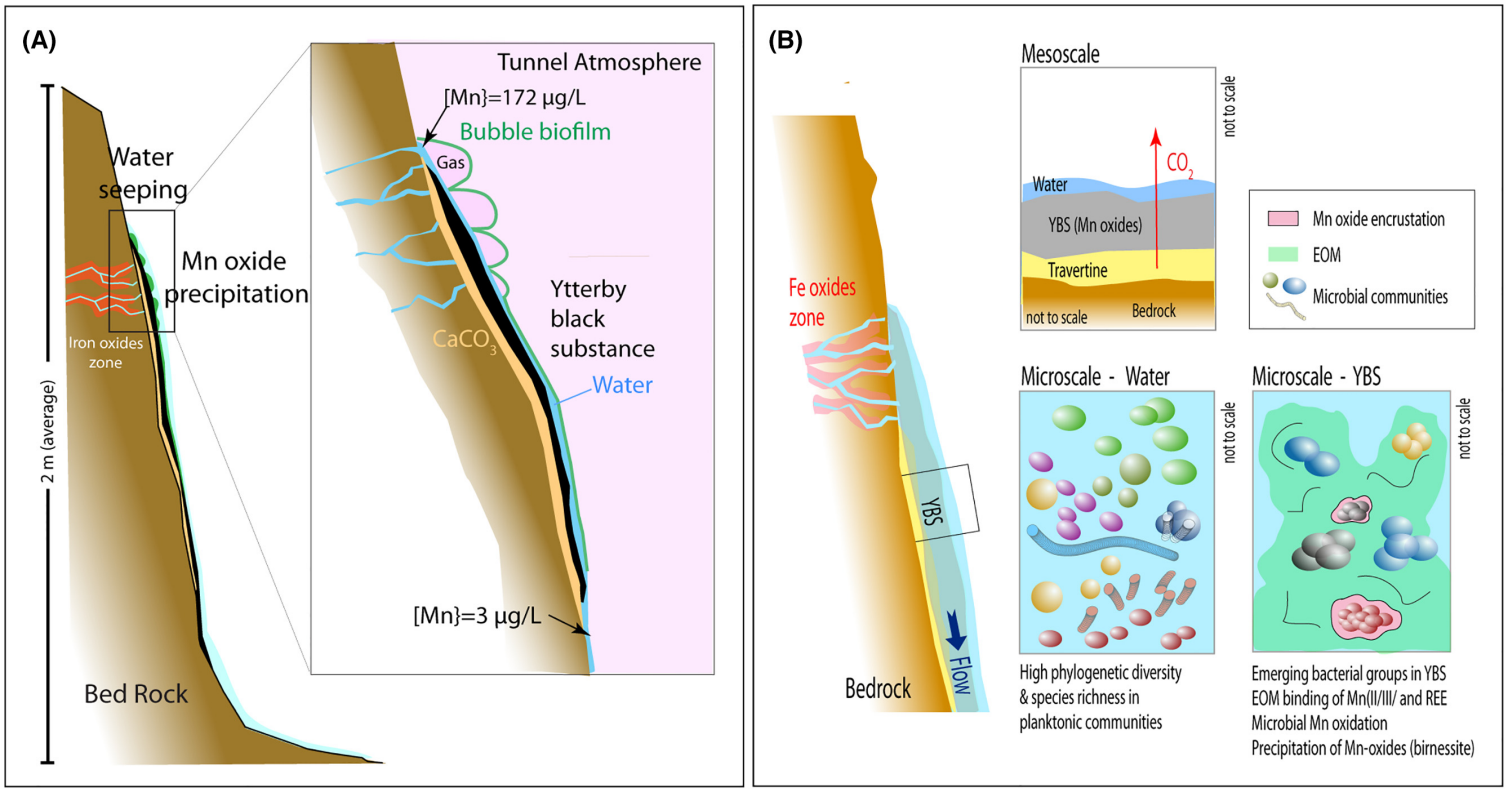
General view of the system showing the YBS biofilm formation and associated metal accumulation in the Ytterby mine tunnels. **(A)**, The fracture feed water contains Mn but almost no Fe. The Mn concentration in the water drops substantially from 172 µg/L at the top or the rock wall (when it leaves the fracture) to only 3 µg/L after it has passed through the biofilms down the approxiamately 2 m tall rock wall. **(B)**, Water seeping from the bedrock fractures equilibrates with the tunnel atmosphere over time, which leads to CO_2_ degassing and travertine precipitation (Pentecost 2005). The water containing Mn(II/III) and REE hosts communities with high phylogenetic diversity and species richness. Planktonic populations settle to produce a thin biofilm at the rock and/or travertine surface. Mn(II/III) and REEs are progressively bound to the organic matter in the biofilm (cells and extracellular organic matter, EOM). The epilithic microbial communities oxidize the trapped Mn(II) and subsequently precipitate Mn(III/IV) oxides. The high binding efficiency of the combined organic and mineral substrates will sorb additional Mn(II/III), REE, other trace elements as well as Ca, Mg. Prokaryotic communities that are adapted to this environment (no light, low temperature and high level of heavy metals) will then thrive and become enriched within the biofilm.

The slightly basic pH of the fracture water (around 8) is just within the range that allows for abiotic Mn(II) oxidation by O_2_, but the reaction is slow, considerably slower than microbe-mediated oxidation. It is therefore expected that microorganisms take advantage of these conditions. A general view of the Mn-oxide producing ecosystem in the Ytterby mine tunnels is shown in Fig. 7. When the water reaches the fully oxidized tunnel there is no Fe left but a Mn concentration of 172 µg/L. A comparison of elemental concentrations in FW_YBS_T (sampled at the top of the rock wall as the water emerges from the fracture), to those of FW_YBS_B (sampled at the bottom of the rock wall after it has passed through the YBS), shows that the Mn(II) concentration drastically drops from 172 µg/L to 3 µg/L. This strong attenuation of aqueous Mn is coupled to enrichment of insoluble oxides in the YBS biofilm, which in turn attracts other metals such as the REE.

Aqueous Mn and REE are progressively bound to the organic matter in the YBS biofilm (cells and EOM) when the water percolates through the YBS biofilm down the 1.5–2 m high rock wall. The epilithic microbial communities oxidize trapped Mn(II) and subsequently precipitate birnessite-type Mn oxides. Catalysis of Mn(II) oxidation by preformed Mn-oxides, i.e. autocatalytical oxidation through the reduction of MnO_2_, is also expected when the initial precipitates are formed (Bargar*et al*. 2005; Tebo *et al*. 2004). The negatively charged Mn oxides then sorb additional REE and Mn(II). For the REE, the ratio (FW_YBS_B/FW_YBS_T) is going from 0.45 for La, being the largest ion in the series (radius 103 pm) to 0.89 for Lu (radius 86 pm) which indicate a preference for the lighter, larger ions. The comparatively high levels of Mn and REE in FW_YBS_T are likely a prerequisite for the differentially abundant taxa in the YBS biofilm to settle and mediate precipitation of Mn-oxides.

### Mn-oxide precipitation within the YBS biofilm

It becomes increasingly clear that the capability of mediating Mn-oxidation is widespread among diverse groups of bacteria and fungi and occurs in a variety of settings such as soil (Bromfield and Skerman 1950; van Veen 1973), freshwater pipelines (Tyler and Marshall 1967; Caspi, Tebo and Haygood 1998), drinking water systems (Breda, Ramsay and Roslev 2017; Marcus *et al*. 2017), freshwater environments (Okazaki *et al*. 1997), marine environments (Nealson 1978; Caspi, Haygood and Tebo 1996), hydrothermal vents (Ehrlich 1983; Cowen, Massoth and Baker 1986) and underground settings (Northup *et al*. 2003; Carmichael *et al*. 2013; Bohu *et al*. 2016). However, Mn-oxidizing archaea have not yet been identified. It also seems increasingly likely that biofilms as a model of microbial organization enhance oxidation.

The key players in the YBS biofilm: *Rhizobiales (e.g. Pedomicrobium)*, PLTA13 *Gammaproteobacteria, Pirellulaceae, Hyphomonadaceae, Blastocatellia* and *Nitrospira* are not detected in the feed water but rather emerge in the YBS biofilm. Similarily to the Fe(II)-oxidizing bacteria that likely found their niche in the low oxygen rock fractures, these bacteria populate an environment where they potentially have less competition and thus can expand more freely. The promotion of Mn-oxides by biofilm formation is previously documented by Nealson and Ford (1980) who observed that a *Bacillus* species had little or no capability of Mn-oxidation as a free floating cell but showed a drastic increase in oxidation rate once the bacterium interacted with solid substrates.

However, the presence and high relative abundance of sequences belonging to the *Mesorhizobium* genus (98.5% similar to an identified Mn-oxidizer; HG932494, Bohu *et al*. 2015) in the FW_YBS_T, in combination with the complete absence of this group in the FW_MM_T and close to absence in the YBS biofilm, is puzzling. It raises the question why this planktonic group of bacteria, likely capable of Mn-oxidation, is not observed in the FW_YBS_B nor in any of the biofilms. The role played by these free floating cells in the formation of this Mn deposit (if any) remains to be determined.

### Petroleum degrading microbes

A high relative abundance of bacteria closely affiliated with sequences capable of degrading hydrocarbons are recurrent through all samples, occasionally in combination of being highly similar to sequences retrieved from metal-rich environments. It is plausible that these species help in the clean-up of residual oil in the mine area, functioning as a natural wastewater treatment where the organic constituents from the petroleum products are consumed and thereby immobilized by the microbial biofilms. Mn-oxidizing bacterial species and sequences retrieved from hydrocarbon contaminated sites are also observed in a previous subterranean study (Carmichael *et al*. 2013). Whether the Mn-oxidizing ability bears a relation to subterranean sites, hydrocarbons and/or heavy metal rich environments is difficult to confirm but it is possible that it is a response to a stressful environment. The high relative abundance of archaea in the top water is noteworthy in itself and the similarity to archaeal gene sequences retrieved from contaminated (e.g. metals, radionuclides) subsurface sites accentuate the need for investigating the role of archaea in these types of environments. Measurements of hydrocarbons in the studied fracture water and in the adjacent mine area are ongoing and more data will be published in a near future.

## CONCLUSIONS

Each of the four subsystems in the underground Mn-oxide producing ecosystem (top and bottom fracture water, YBS biofilm and bubble biofilm) serves as a distinct ecological niche hosting a specific collection of microorganisms. The production of the REE-enriched Mn-oxides is likely driven by the group of differentially abundant bacterial taxa in the YBS biofilm: *Rhizobiales* (e.g. *Pedomicrobium)*, PLTA13 *Gammaproteobacteria, Pirellulaceae, Hyphomonadaceae, Blastocatellia* and *Nitrospira*. These taxa were not detected in the FW_YBS_T which implies that the planktonic population has little impact on the derived biofilm, in which the differentially abundant taxa rather form as a response to water chemistry (Mn and REE), environmental conditions, and possibly high microbial diversity. The gradual sequestration of metals in the biofilm indicates that these newly settled bacterial groups have critical control on the mineral end product (i.e. Mn-oxides) and thus the mobility of metals in the area.

The biofilm and the associated formation of birnessite-type Mn-oxides not only removes cations incorporated in the mineral structure from the water, it also scavenges more than 20 additional trace elements. These trace elements are adsorbed on the birnessite or co-precipitated, and this process contributes to the removal of notably hydrolysable trace elements from the water. The biofilm binds Mn, REE and other trace elements in an efficient, dynamic process, as indicated by substantial depletion of these metals from the fracture water as it passes through the Mn deposit zone: from the top of the rock wall (as the water emerges from the fracture) to the bottom of the deposit (after it has passed through the Mn precipitates down the 2 m tall rock wall). The Ytterby mine ecosystem thus exemplifies a natural, local water remediation process which occurs as the result of a biologically mediated formation of a reactive Mn-oxide phase. To learn more about these processes, our findings here are currently completed with cultivation based studies. The ongoing work involves microstructural characterizations of Mn phases produced *in vitro* by microbes isolated from this ecosystem.
